# Overexpression of SCYL1 Is Associated with Progression of Breast Cancer

**DOI:** 10.3390/curroncol29100544

**Published:** 2022-09-24

**Authors:** Aiqin Sun, Xianyan Tian, Wannian Yang, Qiong Lin

**Affiliations:** School of Medicine, Jiangsu University, Zhenjiang 212013, China

**Keywords:** SCYL1, breast cancer, prognosis, proliferation, migration

## Abstract

SCYL1 is a pseudokinase and plays roles in cell division and gene transcription, nuclear/cytoplasmic shuttling of tRNA, protein glycosylation, and Golgi morphology. However, the role of SCYL1 in human breast cancer progression remains largely unknown. In this study, we determined expression of SCYL1 in breast cancer by searching the Cancer Genome Atlas (TCGA) and Tumor Immunoassay Resource (TIMER) databases. Meanwhile, we collected breast tumor tissue samples from 247 cases and detected expression of SCYL1 in the tumors using the tissue microarray assay (TMA). Association of SCYL1 with prognosis of breast cancer was determined based on the PrognoScan database. The results have shown that SCYL1 is overexpressed in breast cancer, and the expression of SCYL1 is associated with poor clinical outcomes of breast cancer patients. Furthermore, knockdown of SCYL1 by shRNAs significantly inhibited the proliferation and migration of breast cancer cells. Taken together, our data suggest that SCYL1 is a biomarker for poor prognosis of breast cancer, has a promoting role in breast cancer progression, and is a potential target for breast cancer therapy.

## 1. Introduction

Breast cancer (BC) has surpassed lung cancer as the most commonly diagnosed cancer, with an estimated 2.3 million new cases worldwide in 2020, and it has become the No. 1 “killer” disease in women [1,2]. In the past few decades, surgery and chemo-radiotherapy were the mainstay of treatments for well-confined primary breast tumors [3,4]. However, the efficiency of the treatment was often poor, and post-surgery tumor recurrence and remote metastasis were frequently seen [5,6,7]. Metastasis is responsible for the vast majority of patient deaths in breast cancer and is a great challenge for breast cancer therapy [8]. Currently, molecular targeted therapy has attracted considerable attention; various biomarkers of breast cancer, such as HER-2, CA153, Ki-67, have been identified [9,10]. It still has paramount urgency to establish new biomarkers and therapeutic options, especially in metastatic breast cancer, to improve the survival and prognosis of breast cancer patients.

SCYL1 (SCY1-like pseudokinase 1), also known as NTKL or TEIF, is a catalytically inactive protein kinase belonging to the SCY1-like family, which is highly evolutionarily conserved among eukaryotes and widely expressed [11,12]. It has been demonstrated that the SCYL1 structure mainly contains three domains: an N-terminal pseudokinase domain serving as modulation of active kinases or assembly of signaling pathways, a centrally located seven HEAT repeats domain for Homo-oligomerization and tRNA binding, and a C-terminal segment containing one or more coiled-coil domains for protein-protein interaction [13,14]. Mutations in SCYL1 are associated with CALFAN syndrome, characterized by low γ-glutamyltransferase cholestasis, acute liver failure, and neurodegeneration [15,16,17]. SCYL1 physically interacts with γ-COP and ARF4 and is involved in intracellular trafficking [18]. SCYL1 functions in cell division and gene transcription, nuclear/cytoplasmic shuttling of tRNA, protein glycosylation, and Golgi morphology [19,20,21,22].

Currently, little is known about the role of SCYL1 in cancer progression and metastasis. Early study has showed that centrosomal localization of SCYL1 is associated with centrosome amplification and telomere dysfunction, suggesting SCYL1 is likely to play a role in cancer development [12,23]. SCYL1 was reported to mediate cell cycle progression and cell motility to promote hepatocellular carcinoma tumorigenicity [24]. It has been shown that SCYL1 cooperates with TEX14 and PLK1 to constitute the oncogenic signaling STP axis, which promotes triple-negative breast cancer through downregulation of the tumor suppressor REST [25]. However, another study found that SCYL1 does not regulate REST expression and turnover [26]. Thus, the role of SCYL1 in breast cancer remains unclear.

In this report, we used bioinformatics analysis of data from public datasets to identify SCYL1 expression differences in tumors and normal samples. We also examined the expression of SCYL1 in 247 breast cancer tissues with immunohistochemical (IHC) staining. Then, the PrognoScan database was employed to assess the prognostic significance of SCYL1 in breast cancer. Moreover, we performed in vitro experiments to evaluate the effects of SCYL1 on breast cancer cell proliferation and migration. Therefore, this study aims to investigate whether SCYL1 is abnormally expressed in breast cancer and affects the behavior of breast cancer cells, in order to evaluate the value of SCYL1 in the prognosis of this disease.

## 2. Materials and Methods

### 2.1. Reagengts

Antibodies for SCYL1 (A6735) and Actin (A5441) were purchased from Abconal Technology (Wuhan, China) and Sigma-Aldrich (St. Louis, MO, USA), separately. Anti-Mouse (31,430) and anti-Rabbit (31,460) HRP-conjugated secondary antibodies were from ThermoFisher Scientific (Waltham, MA, USA). The shRNA oligos for SCYL1 were produced by Sangon Biotech Company (Shanghai, China). Transwell chambers (3422) were purchased from Corning Inc (Corning, NY, USA). Puromycin was purchased from Biotopped (Beijing, China). The DAB kit for IHC was from Zhongshan Jinqiao Biotechnology (Beijing, China). The SABC-POD kit and Mayor’s hematoxylin were purchased from Boster Biological Technology (Wuhan, China). Human breast cancer cell lines MDA-MB-231 and MCF-7 were obtained from ATCC (Manassas, VA, USA).

### 2.2. Gene Expression Analysis

We applied the “Gene-DE” module of TIMER2.0 (http://timer.cistrome.org/, accessed on 25 April 2022) to obtain the differential expression data of SCYL1 between the primary tumor and the normal samples in different tumor types from TCGA (The Cancer Genome Atlas) databases. The statistical significance evaluated using the Wilcoxon test is annotated by the number of stars (*p*-value < 0.05).

The raw data of gene expression in breast cancer and corresponding clinical information were downloaded from the TCGA website (https://portal.gdc.cancer.gov/, accessed on 21 April 2022), including 942 breast cancer specimens and 95 normal breast specimens. Gene expression and clinical data were decompressed and merged into a matrix file by Perl software, and differentially expressed analysis of SCYL1 was performed using the R/Limma package.

### 2.3. The Prognostic Value of SCYL1 Analysis

We analyzed the correlation between SCYL1 expression and prognosis of BC patients based on the PrognoScan platform (http://gibk21.bse.kyutech.ac.jp/PrognoScan/index.html, accessed on 18 May 2022) [27]. The cohort GSE1456-GPL97 was used to explore the prognostic value of SCYL1, such as overall survival (OS), disease-specific survival (DSS) and relapse free survival (RFS). Patients with BC were classified into high expression and low expression groups based on the optional cut-off value of SCYL1.

### 2.4. Human Tissue Specimens and Patient Information

To make the BC tissue microarrays, we collected 106 triple-negative breast cancer (TNBC) and 141 non-triple-negative breast cancer (NTNBC) samples from the Department of Breast Surgery, Affiliated People’s Hospital, Jiangsu University. We used these tissue samples with informed consent and the Hospital Institutional Review Board approval. In the meantime, we got their corresponding paired adjacent normal tissues (80 to TNBC, 93 to NTNBC) as the control.

### 2.5. Immunohistochemistry (IHC) Staining

The staining procedures were previously described [28,29]. The antibody specific for investigation of the level of SCYL1 expression in the tumor was described above. The scoring criteria: the total scores of IHC staining were calculated based on staining percentage scores (classified as 1 (1–25%), 2 (26–50%), 3 (51–75%), 4 (76–100%)) and staining intensity scores (scored as 1: light-yellow, 2: yellow, 3: brownish-yellow, 4: dark brown). IHC-score ≥ 4 was interpreted as positive.

### 2.6. Knockdown of SCYL1 by the shRNA

The breast cancer cell lines MDA-MB231 and MCF-7 cells were cultured in DMEM with 10% fetal bovine serum, 100 units/mL penicillin and streptomycin at 37 °C with 5% CO_2_. The SCYL1 shRNAs (1#:GCTACACCAGATCGTGAAAGC, 2#:CGCCTTCGAGTTCGGCAATGC) were sub-cloned into the lentiviral shRNA expression vector PLKO.1 (Addgene) via AgeI/EcoRI sites.

Establishment of stable cell line expressing interest shRNA was performed as described previously [30]. Briefly, lentiviral particles were packaged in HEK293T cells. Viral supernatants were added to MDA-MB-231 and MCF-7 cells for 24 h, and stably transduced populations were screened by administration of 2 μg/mL puromycin. Immunoblotting analysis was preformed to detect the expression of SCYL1 by using an anti-SCYL1 antibody.

### 2.7. Cell Lysates Preparation and Immunoblotting

The BC cells were washed once with ice-cold PBS and extracted using ice-cold Mammalian cell lysis buffer supplemented with proteinase inhibitors aprotinin and leupeptin (Biotopped, Beijing, China). Proteins were denatured and separated on 8% NuPAGE Bis-Tris SDS gels. Following electrophoresis, separated proteins were transferred onto PVDF membranes (Millipore, MA, USA) and sealed with 1% BSA. Then, the antibody was sequentially hatched with membranes and finally detected by Western Lightning immunoblot Kit (Beyotime, Nantong, China).

### 2.8. Cell Proliferation Assay

3–5 × 10^4^ BC cells were cultured in 12-well plates for indicated times. Then, cells were counted at days 2, 3 and 4 using a hemocytometer under the phase microscope. The increased cell number since seeded was used to assess cell proliferative activity. Each cell counting was performed at least 3 times.

### 2.9. Cell Migration Assays

For the analysis of the migration ability of cells, a wound healing assay and the transwell assay were conducted. (1) For the wound-healing assay, 2–5 × 10^5^ cells were plated in a 12-well plate. When the BC cells were grown to nearly confluent cell monolayers, the scratch wound was created with a sterile pipette tip in the cell monolayer. After scratching, the wound was photographed under a phase microscope at 0 and 24 h. (2) For the transwell migration assay, the BC cells were made into cell suspension in the serum-free medium and inoculated into the upper chamber. A 10% FBS-containing DMEM medium without cells was placed into the bottom chamber as a chemoattractant. The migrated cells that adhered to the bottom surface of the membrane were fixed with 4% paraformaldehyde for 30min, stained with crystal violet (Beyotime, Nantong, China) for 10 min, and photographed.

### 2.10. Statistical Analysis

More than two independent assays were completed for each analysis. Data are displayed as mean ± standard deviation (SD). Statistical difference was analyzed using Student’s *t* test or one-way ANOVA. *p* value less than 0.05 was considered statistically significant. *: *p*-value < 0.05; **: *p*-value < 0.01; ***: *p*-value < 0.001.

## 3. Results

### 3.1. Expression of SCYL1 mRNA Is Increased in Breast Cancer Patients Than Normal Controls

**Figure 1:**
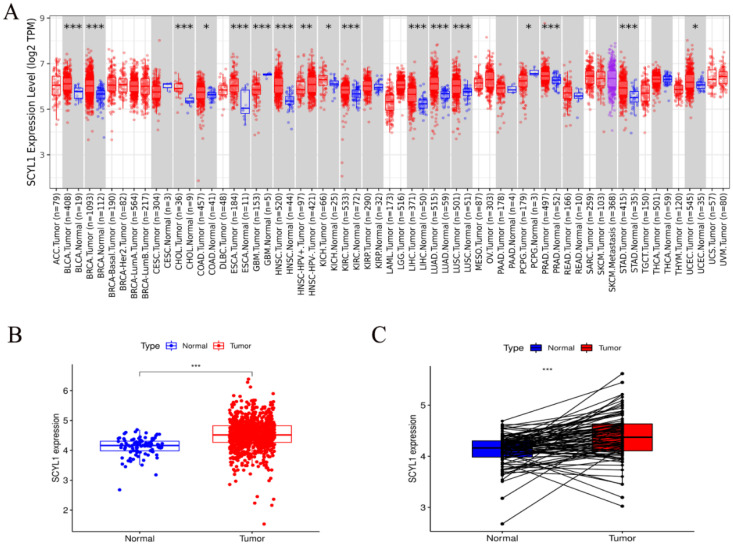
Analysis of SCYL1 expression in breast cancer in the breast cancer database. (**A**) The expression level of SCYL1 in different types of tumor tissues and normal tissues was assessed in TIMER2.0 database. (**B**) Based on the information of 95 normal and 942 tumor samples from Breast Cancer patients in The Cancer Genome Atlas (TCGA) database, the differential expression of SCYL1 was analyzed. (**C**) The pairing difference analysis of SCYL1 expression between the tumor and normal tissues from TCGA database. *: *p*-value < 0.05; **: *p*-value < 0.01; ***: *p*-value < 0.001.

In order to explore the discrepancies of SCYL1 expression in human cancers, the SCYL1 expression in different tumors and normal tissues of multiple cancer types were analyzed using the RNA-seq data in TCGA database through the TIMER2.0. The results revealed that SCYL1 expression was significantly upregulated in BLCA (bladder urothelial carcinoma), BRCA (breast invasive carcinoma), CHOL (cholangiocarcinoma), COAD (colon adenocarcinoma), ESCA (esophageal carcinoma), HNSC (head and neck squamous cell carcinoma), KICH (kidney chromophobe), KIRC (kidney renal clear cell carcinoma), LIHC (liver hepatocellular carcinoma), LUAD (lung adenocarcinoma), LUSC (lung squamous cell carcinoma), PRAD (prostate adenocarcinoma), STAD (stomach adenocarcinoma), and UCEC (uterine corpus endometrial carcinoma). Nevertheless, SCYL1 expression was significantly reduced in GBM (glioblastoma multiforme) and PCPG (pheochromocytoma and paraganglioma) than in normal controls (Figure 1A).

Particularly, the SCYL1 expression in patients with BRAC breast cancer was detected in TCGA database. As shown in Figure 1B, the expression of SCYL1 in breast cancer was significantly increased compared with that of the paracancer normal tissues. The paired differential analysis showed that SCYL1 expression in BRAC tissues was higher than that in corresponding paired non-cancerous adjacent tissues (Figure 1C). These results suggest that SCYL1 may play a role in the progression of breast cancer.

### 3.2. SCYL1 Is Abundantly Expressed in Breast Cancer Tumor Tissues

**Figure 2:**
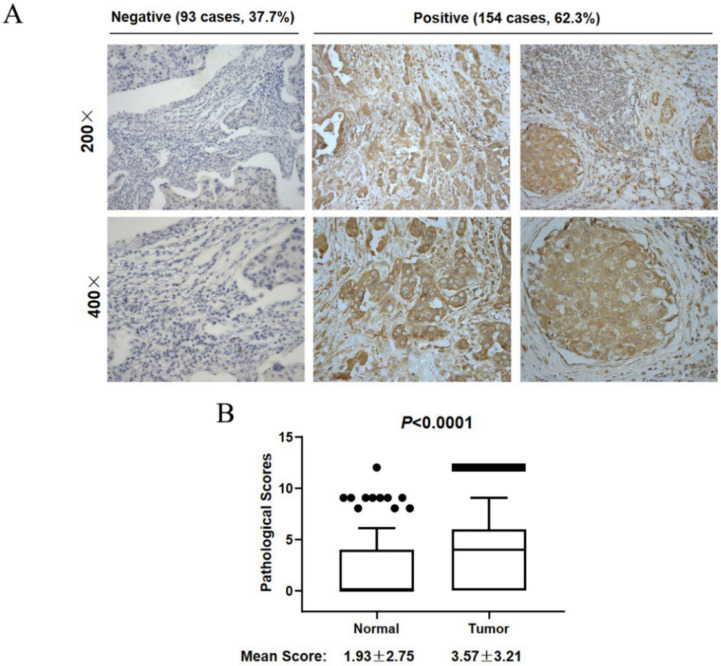
SCYL1 is highly expressed in breast cancer. (**A**) IHC staining of SCYL1 in BC tumor samples. Both SCYL1 negative (the left two panels) and positive (the right four panels) tumor samples are shown. (**B**) Expression of SCYL1 in BC tumor tissue is significantly higher than in their adjacent normal tissue. The average scores and standard deviations of IHC staining of SCYL1 in both BC tumors (Tumor) and their adjacent normal tissue (Normal) are shown in the figure. The dots and bar above the boxplot represent outliers.

To further understand the role of SCYL1 in breast cancer development, IHC staining was performed using a tissue microarray assay (TMA) consisting of 247 breast cancer samples. As shown in Figure 2A, the average IHC staining score of SCYL1 in the breast cancer tumor tissues is 3.77 ± 3.21, compared to the score of normal tissues at 1.93 ± 57.75, indicating that expression of SCYL1 in the breast cancer tumors is significantly higher than in their adjacent normal tissues (*p* < 0.0001). According to the TMA assay, the results showed that 154/247 breast cancer patients (62.3%) were strongly stained with anti-SCYL1 (the right 4 panels, Figure 2B), and 93 tumor samples (37.7%) had weak or no staining (the left 4 panels, Figure 2B). These IHC results confirmed that SCYL1 is upregulated in breast cancer.

### 3.3. Overexpression of SCYL1 Is Associated with a Poorer Prognosis in Breast Cancer

**Figure 3:**
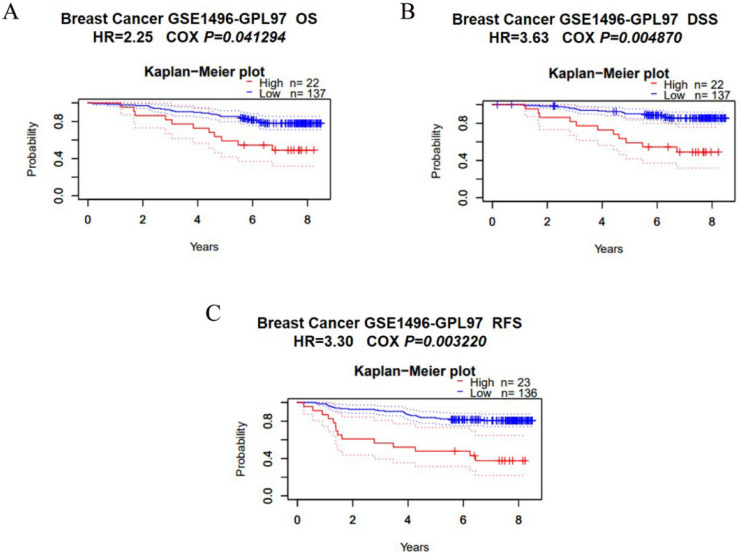
Expression of SCYL1 is associated with poor clinical prognosis. (**A**–**C**) the correlation between SCYL1 expression and prognostic value of BC was obtained through the PrognoScan. OS, overall survival; DSS, Disease special survival; RFS, Relapse free survival.

Both the bioinformatics results and IHC staining results of our in-house cases suggested SCYL1 is significantly higher expressed in patients with breast cancer. Subsequently, we investigated whether SCYL1 expression was correlated with prognosis in breast cancer patients. The impact of SCYL1 expression to survival data was evaluated using the PrognoScan database, we found high expression of SCYL1 was associated with worsened prognoses in breast cancer patients as measured by overall survival (OS), disease special survival (DSS), and relapse free survival (RFS) (Figure 3). Thus, these data clearly demonstrated that SCYL1 may be applied as a valuable biomarker for poor prognosis and might have an important role in the progression of breast cancer.

### 3.4. Downregulation of SCYL1 Inhibits Breast Cancer Cell Proliferation

**Figure 4:**
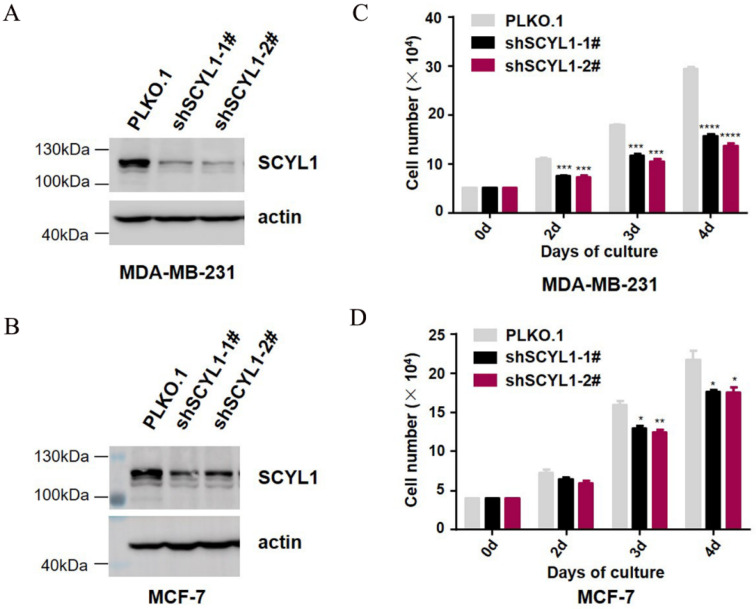
Knockdown of SCYL1 partially suppresses proliferation of BC cells. Endogenous SCYL1 was depleted by lentiviral vector-loaded SCYL1 shRNA for 48 h and detected by immunoblotting with anti-SCYL1 from the cell lysates. The effect of SCYL1 knockdown on cell proliferation was quantified by counting the cell number under a phase microscope with a hemocytometer. The data used for quantification were from three independent experiments. (**A**,**B**) SCYL1 expression at protein levels in MDA-MB-231 and MCF-7 cells. (**C**,**D**) The effect of SCYL1 knockdown on MDA-MB-231 and MCF-7 cell proliferation. *: *p*-value < 0.05; **: *p*-value < 0.01; ***: *p*-value < 0.001; ****: *p*-value < 0.0001.

To further characterize the biological functions of SCYL1 in the progression of breast cancer, lentiviral-mediated shRNAs were used to silence endogenous SCYL1expression in breast cancer cell lines, including MDA-MB-231 and MCF-7. A shRNA sequence with no targeting effect served as the negative control, and the efficiency of knockdown was confirmed by immunoblotting analyses (Figure 4A,B and Supplement Figure S1). The cell viability was analyzed by cell counting assay after downregulation of SCYL1, both MDA-MB-231 and MCF-7 cells showed a significant decrease in cell numbers (Figure 4C,D). These results revealed that knockdown of SCYL1 significantly inhibited cell proliferation in breast cancer.

### 3.5. Downregulation of SCYL1 Inhibits Breast Cancer Cell Migration

**Figure 5:**
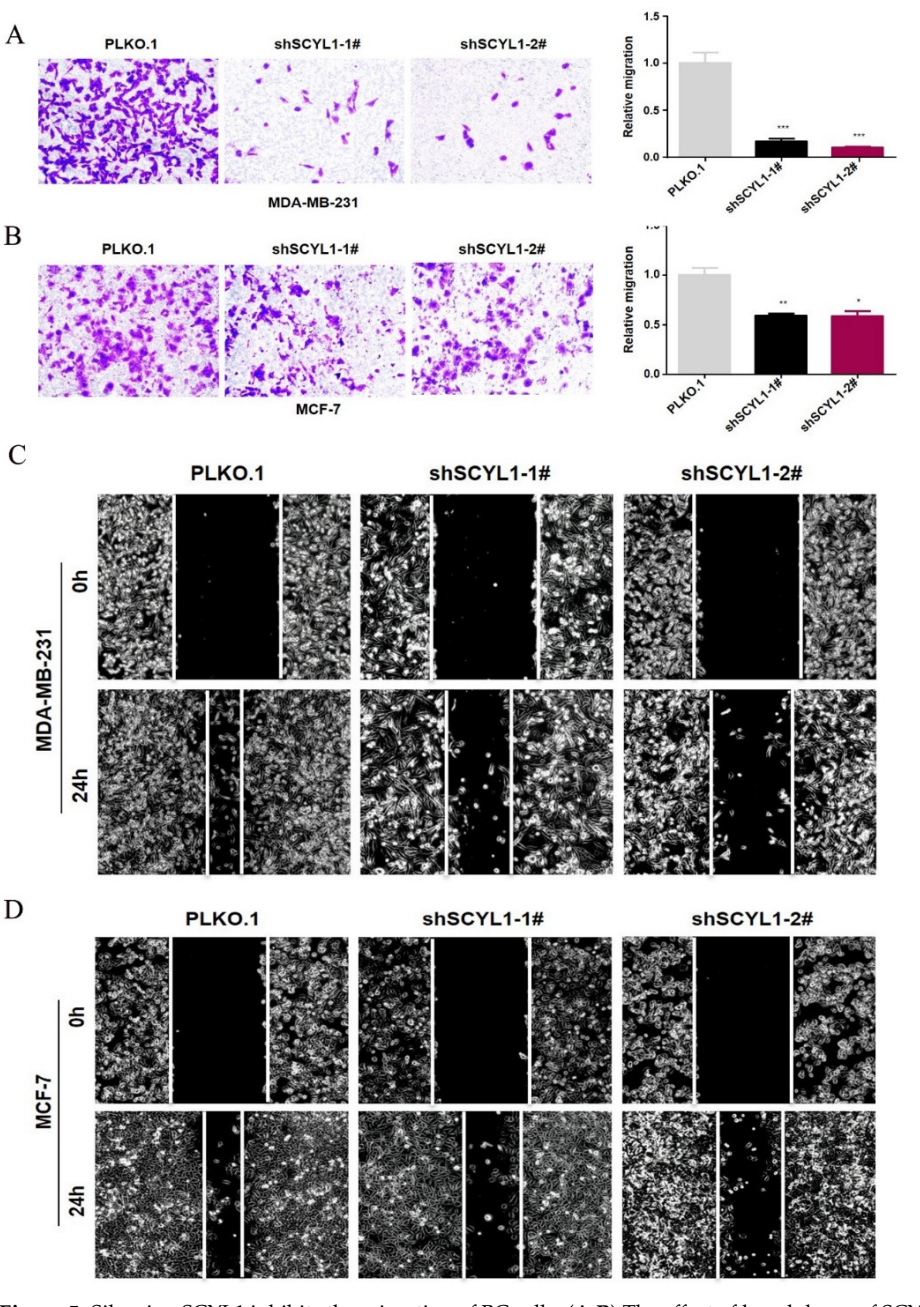
Silencing SCYL1 inhibits the migration of BC cells. (**A**,**B**) The effect of knockdown of SCYL1 on cell migration determined by the transwell assay; statistical results of invaded cells in three randomly selected fields in each sample; original magnifications ×200. (**C**,**D**) The wound healing assay of the effect of SCYL1 knockdown on the breast cancer cell migration; original magnifications ×100. *: *p*-value < 0.05; **: *p*-value < 0.01; ***: *p*-value < 0.001.

Next, we carried out the transwell migration assay to determine the effect of SCYL1 knockdown on breast cancer cell migration in both shSCYL1 cell lines. As shown in Figure 5A,B, the migration ability of MDA-MB-231 and MCF-7 cells in the shSCYL1 group decreased by different level, compared with the control group. Consistently, wound healing assays showed that wound closure speed of MSA-MB-231 and MCF-7 cells were significantly reduced after knockdown of SCYL1 (Figure 5C,D). Based on these results, we inferred that silencing endogenous SCYL1 expression can suppress the migration of breast cancer cell lines MDA-MB-231 and MCF-7.

## 4. Discussion

In this study, we first observed the expression of SCYL1 is elevated at mRNA levels in 14 types of tumor tissues, suggesting that SCYL1 might be an important pro-oncogenic protein in these tumor types. Through mining TCGA datasets data, hyperexpression of SCYL1 was observed in breast cancer tissues compared with adjacent normal tissues. IHC staining of 247 BC tumor samples has shown that SCYL1 was overexpressed in 62.3% of BC tumors. More importantly, investigation of the impact of SCYL1 on BC using PrognoScan analyses revealed that increased SCYL1 levels significantly correlated with a short OS, DSS, and RFS. These findings supported that high SCYL1 expression may effectively predict breast cancer prognosis.

Dysregulation of gene expression may lead to tumor initiation and progression [31]. We used the lentiviral shRNA plasmid vector that knockdown SCYL1 to study the role of SCYL1 in breast cancer cell lines. Downregulation of SCYL1 in breast cancer cells severely inhibits cell proliferation. The transwell migration and wound healing assays showed that downregulation of SCYL1 in breast cancer cells impaired their abilities in migration. These results demonstrated that SCYL1 has a role in promoting breast cancer cell proliferation and migration, the two cellular processes in cancer progression [32].

An important process thought to be regulated by SCYL1 is vesicular transport of Golgi apparatus [18,22,33]. Specifically, the Golgi apparatus was shown to be enlarged and fragmented, with an increased volume in SCYL1 knockdown cells [21]. The role of Golgi apparatus in tumorigenesis has been brought to attention in recent years. Accumulating evidence has shown that Golgi acts as a hub for signaling molecules in cancer progression [34,35,36], which is involved in numerous cellular processes, such as cancer cell dissociation, cell-matrix adhesion, tumor angiogenesis, migration, and invasion [37,38,39]. Our studies clearly indicate that SCYL1 functions in progression of breast cancer; it is possible that SCYL1 promotes tumorigenesis of breast cancer via regulating vesicle trafficking and structure of Golgi.

Up to now, studies on the role of SCYL1 in breast cancer progression are still rare. Karlin et al. demonstrated SCYL1 as one of the components of the oncogenic STP axis, which was a driver of triple-negative breast cancer by suppressing REST protein levels via degradation [25]. Contrary to that data, a recent study found RNAi–mediated knockdown of SCYL1 in MDA-MB-231 cells did not alter REST steady-state level and turnover; thus, SCYL1 is dispensable for the down-regulation of REST [26], suggesting in some cases the function of SCYL1 in tumor progression may not be dependent on degradation of REST. In our studies, the major role of SCYL1 seems promoting BC tumor cell proliferation and migration, whether SCYL1 promotes BC through degradation of the REST warrants further investigations.

In conclusion, our findings indicate that SCYL1 is overexpressed in breast cancer and expression of SCYL1 is associated with poor prognosis of breast cancer. In addition, knockdown of SCYL1 dramatically inhibits proliferation and migration of breast cancer cells, suggesting a promoting role of SCYL1 in breast cancer progression. Further study is needed to better understand the mechanisms by which SCYL1 regulates breast cancer progression.

## Data Availability

The data needed to evaluate this work are all included in the manuscript and are available upon reasonable request.

## Supplementary Materials

The following supporting information can be downloaded at: https://www.mdpi.com/article/10.3390/curroncol29100544/s1, Figure S1: Uncropped Western Blot Figures.
