# Supplementary figures and images for: Overexpression of SCYL1 Is Associated with Progression of Breast Cancer
Source: Curr Oncol. 2022 Sep 24;29(10):6922–32. doi: 10.3390/curroncol29100544 (PMC9600755; doi:10.3390/curroncol29100544)

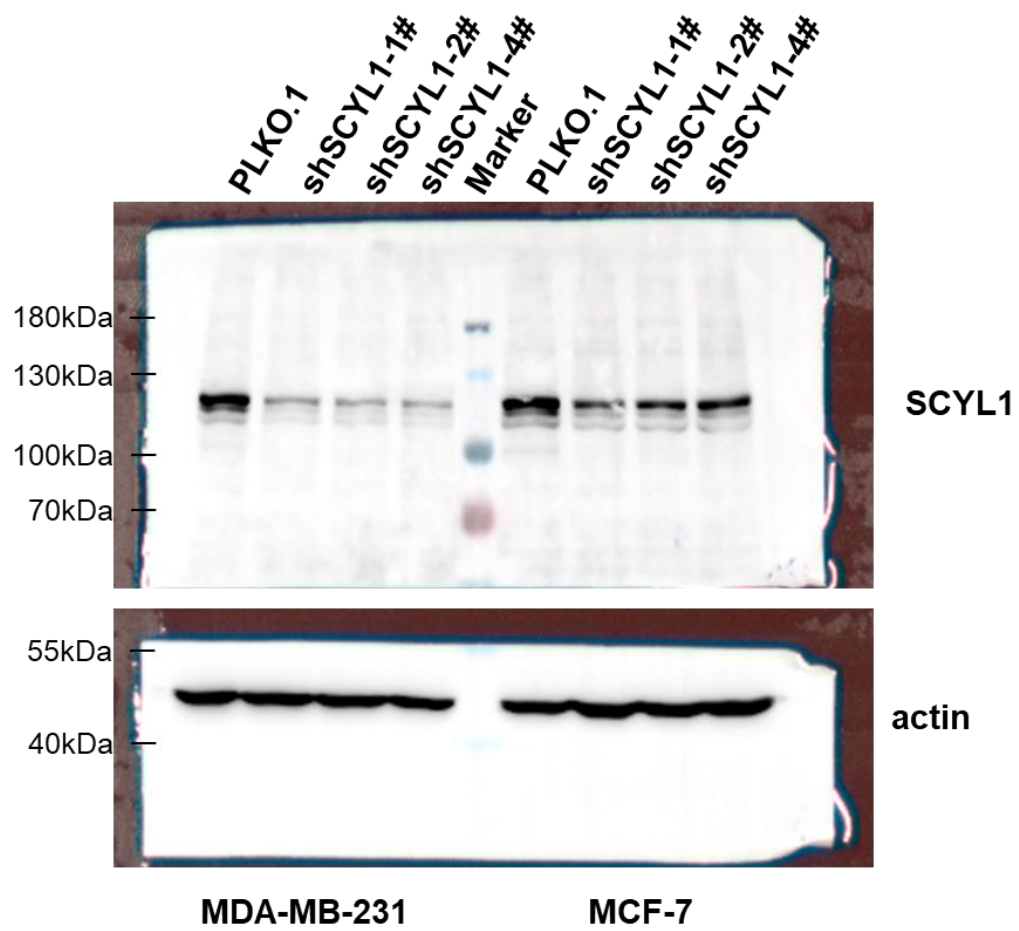

**Figure S1.** Uncropped Western Blot Figures.

Supplement: Supplementary file 1 [file curroncol-29-00544-s001.zip › curroncol-1882688-supplementary.pdf]
